# Composite Contrast Enhancement of Hydrogel-Based Implants for Photon-Counting Computed Tomography Studies

**DOI:** 10.3390/gels10120807

**Published:** 2024-12-08

**Authors:** Evgeniya V. Suslova, Denis A. Shashurin, Konstantin I. Maslakov, Stepan Yu. Kupreenko, Tatyana O. Luneva, Oleg S. Medvedev, Georgy A. Chelkov

**Affiliations:** 1 Department of Chemistry, Lomonosov Moscow State University, Leninskie Gory 1 Bld. 3, 119991 Moscow, Russiakupreenko@physics.msu.ru (S.Y.K.); luneva.tatiana2016@yandex.ru (T.O.L.); 2 Faculty of Medicine, Lomonosov Moscow State University, Lomonosovsky Ave. 27 Bld. 10, 119991 Moscow, Russia; oleg.omedvedev@gmail.com; 3 Research and Educational Resource Center for Immunophenotyping, Digital Spatial Profiling and Ultrastructural Analysis Innovative Technologies, RUDN University, 6 Miklukho-Maklaya St., 117198 Moscow, Russia; 4 Joint Institute for Nuclear Research, Joliot-Curie 6, 141980 Dubna, Russia; chelkov@jinr.ru

**Keywords:** lanthanides, gadolinium, polyacrylamide, contrast agent, implant, phantom, photon-counting computed tomography

## Abstract

Hydrogels have a wide range of medical applications, including use within implantable systems. However, when used in implants, their visibility under conventional medical imaging techniques is limited, creating safety risks for patients. In the current work, we assessed the possibility of enhancing hydrogels using Ln-based contrasting agents to facilitate their visualization in photon-counting computed tomography (PCCT). The contrast enhancement of gelatin, polyacrylamide (PAM), and silicone shells of implants was assessed. A novel synthetic route for producing cross-linked nanosized Ln_2_O_3_ with polyacrylamide was proposed and discussed in detail. Several prototypes of silicone implants, including silicone shell and gelatin or PAM filling with different combinations of contrasting agents, were produced and assessed in phantom PCCT studies.

## 1. Introduction

Hydrogels have a wide range of medical applications [1]. Their tunable physico-chemical properties, relative biocompatibility [2], stability, and biodegradability make them suitable for use as pharmaceutical substances, drug delivery systems [3], inner fillings of implantable systems, or even as shell-less implantable sensors [4].

Despite this, hydrogels have a significant disadvantage that introduces risk when used for medical purposes, especially as implant materials. They are only partially visible through conventional medical imaging methods such as ultrasound, X-ray, or magnetic resonance imaging (MRI) [5,6]. This complicates the regular assessment of a gel-filled implant and the timely diagnosis of its malfunctions, such as ruptures with extracapsular leakage of the inner material [7].

One potential solution for this can be the addition of compounds with high X-ray attenuation coefficients to hydrogels, enhancing their visibility with roentgenological and some other imaging methods [8,9]. The compound most commonly used as a contrasting agent for X-ray imaging is iodine [10]. Hydrogels or similar biomaterials can be contrasted by iodinated fragments of tyrosine residues [11], wholly aliphatic iodinated polyesters [12], iodinated polyurethane [13], or covalently iodinated polymethacrylate [14], facilitating their visualization in biomedical studies by computed tomography (CT). Hydrogels can also be contrasted by barium, bismuth sulfate [15], or even radioactive isotopes such as ^111^In^3+^, making them suitable for complex diagnostic studies using single-photon emission computed tomography (SPECT) and positron emission tomography (PET) [16].

The modification of hydrogels with elements possessing high X-ray attenuation coefficients [17] proves particularly effective when coupled with advanced diagnostic methods such as photon-counting computed tomography (PCCT) combining CT with elements of X-ray spectroscopy [18]. It provides a visualization of the inner structures of study objects, accounting not only for their average X-ray attenuation but also their unique X-ray attenuation spectra, giving insights into their chemical composition and making it possible to distinguish between areas with comparable roentgenological density but different elemental contents [19,20]. PCCT has been extensively used in biomedical research and is now being introduced into clinical practice. Over 30 clinical trials assessing PCCT capabilities in comparison with conventional (energy-integrative) CT scanners in different indications have been published [21,22]. PCCT allows the simultaneous use of multiple contrast agents (CAs), including elements not present in the human body (i.e., lanthanides La, Nd, Eu, Gd, Dy, Ho, Er, Yb, Lu [23,24,25,26,27,28], Hf [29], Ta [24], Bi [30], W [30], and Au [31]) for separate visualizations of different contrasted structures within a single assessment. This means that the implementation of such contrasting agents into the filling of the implants can not only enhance their visualization but also enable differentiation from components of the same or different implants, which can be critical for assessing their condition.

The CAs of special interest for PCCT studies are lanthanides (Ln). This group has similar chemical properties but different K-edge energies, leading to different X-ray attenuation spectra. So, the same synthetic processes can be used to produce different CAs and contrast-enhanced materials for subsequent simultaneous visualization by PCCT [23].

The most common CA belonging to lanthanides is Gd, which is actively used as a contrast enhancement in MRI studies [32] and is probed as a reserve CA for CT studies in patients with severe allergic reactions to standard iodinated contrasts [33]. It is also considered promising for dual-mode CT/MRI studies [34]. Gd contrasts used in clinical practice typically have the structure of chelate complexes of Gd^3+^ with polycarbonate acids [35]. More recently, considerable advances have also been made in developing Gd-based CAs in the form of nanoparticles, coated or stabilized by proteins, saccharides, lipids, or inorganic matrices consisting of carbon or SiO_2_ [36]. CA nanoparticles demonstrate comparable or higher contrasting properties in MRI or CT studies compared to molecular contrasts [37,38]. Such CAs are also considered to have low cytotoxicity [39,40]. This is attributed to their high chemical stability and low release of free Gd^3+^ ions which are considered the primary mechanism of Gd-induced toxicity [41]. However, it should be noted that most of the studies evaluated Gd^3+^ concentrations below 100 mg·L^−1^, while commercially available Gd contrasts (i.e., gadavist [42]) are administered in doses leading to blood Gd^3+^ concentrations exceeding 200 mg·L^−1^. Gd-based nanoparticle CAs may have different mechanisms of toxicity independent of the release of Gd^3+^ [43], indicating the need for further research on their possible toxic effects in different modes of administration. Other lanthanides have been probed and studied as potential CAs to a much lesser extent.

The goal of the current work was to assess the possibility and effectiveness of the contrast enhancement of prototype implants based on gelatin or polyacrylamide (PAM) fillings and silicone shells by Ln_2_O_3_ (Ln = La, Gd, and Yb). We assessed the possibility of introducing Ln_2_O_3_ into the PAM matrix during the co-polymerization of monomers or into the commercially available bi-component silicone during molding. We developed novel synthetic routes, making it possible to (1) decrease the size of Ln_2_O_3_ nanoparticles and (2) establish the covalent bonding of these particles to PAM to ensure stability and uniform distribution of the contrasts. The resulting compounds were assessed by physico-chemical methods and tested in PCCT studies of the prototype implants.

## 2. Results and Discussion

### 2.1. Calibration of Material Determination by PCCT and Material Choice for Contrasted Implants

Hybrid pixel detectors used in PCCT have a limited number of energy thresholds and relatively low energy resolution. Consequently, the resulting X-ray attenuation spectra significantly differ from those produced using theoretical data or more precise experimental methods [44]. This requires calibration of the PCCT device and its analysis software for the determination of specific materials or chemical elements anticipated in the study objects. During calibration, a unique attenuation profile for selected energy thresholds and their dependency on concentration is created for each material. These profiles are then compared with the actual profile of each voxel of the 3D reconstruction, and the material with the most fitting profile is defined and quantified [45].

**Figure 1:**
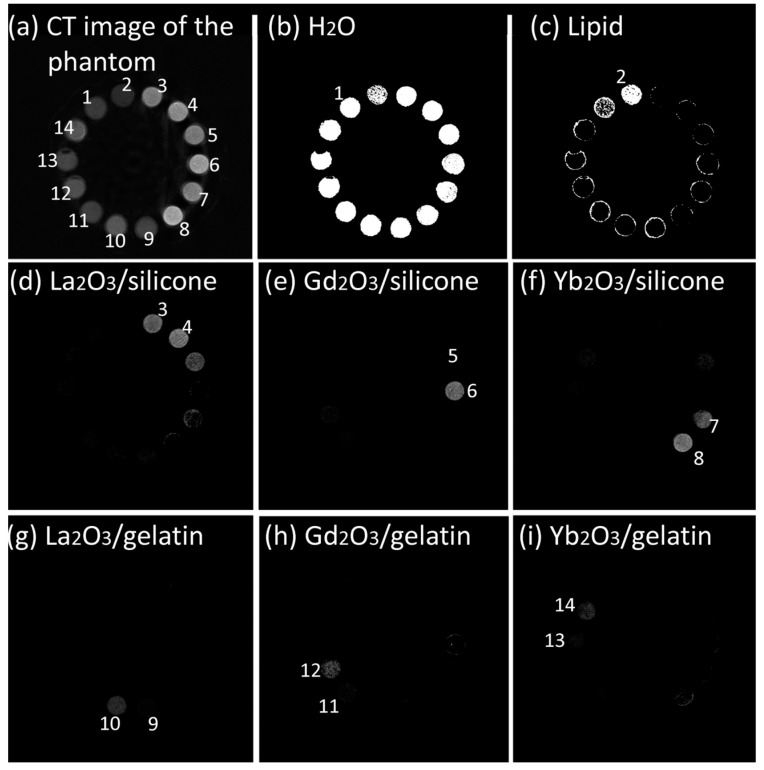
(**a**) Reconstructed transversal slice of calibration phantom where 1 is H_2_O, 2 is lipid, 3 and 4 are La_2_O_3_/silicone with 10 and 40 mg·mL^−1^, 5 and 6 are Gd_2_O_3_/silicone with 10 and 40 mg·mL^−1^, 7 and 8 are Yb_2_O_3_/silicone with 10 and 40 mg·mL^−1^, 9 and 10 are La_2_O_3_/gelatin with 10 and 40 mg·mL^−1^, 11 and 12 are Gd_2_O_3_/gelatin with 10 and 40 mg·mL^−1^, and 13 and 14 are Yb_2_O_3_/gelatin with 10 and 40 mg·mL^−1^. (**b**–**i**) Results of application of material determination criteria for determination of H_2_O (**b**), lipid (**c**), La_2_O_3_/silicone (**d**), Gd_2_O_3_/silicone (**e**), Yb_2_O_3_/silicone (**f**), La_2_O_3_/gelatin (**g**), Gd_2_O_3_/gelatin (**h**), and Yb_2_O_3_/gelatin (**i**).

Our pilot studies revealed that simple calibration using only water solutions of the contrasting elements was insufficient for the accurate determination of Ln_2_O_3_-containing silicones due to the impact of Si on the X-ray attenuation spectra of the Si-containing materials [46]. Therefore, for implant prototypes incorporating both contrasted gelatin fillings and silicone shells, separate calibration profiles of Ln-containing gelatins and silicones were produced using a phantom, including two samples of each material in two concentrations (Figure 1a). The concentrations were chosen based on our previous studies confirming that the lowest range for Ln detection and quantification by PCCT using reference samples is 2.5–5 mg·mL^−1^ [23,25]. Thirty reconstructed transversal slides were used. The analysis of the calibration applied reversely to the reconstruction phantom (Figure 1b–i) confirmed that all Ln_2_O_3_-containing gelatins could be determined accurately, as well as Gd_2_O_3_- and Yb_2_O_3_-containing silicones. La_2_O_3_-containing silicone demonstrated determination artifacts, likely due to the low K-edge energy of La diminishing the La component in the X-ray attenuation spectrum of the contrasted silicone (Figure 1d). This material was excluded from further studies.

Studies using PAM-based implant prototypes not requiring the determination of contrasted silicones have been conducted with simper calibration using La(NO_3_)_3_ and Gd(NO_3_)_3_ solutions. The calibration process also included Ca(C_2_H_3_O_2_)_2_ solutions to support the determination of Ca structures (i.e., bones) in future studies, as well as H_2_O and lipid samples mandated by the calibration algorithm.

### 2.2. Preparation and Phantom PCCT Studies of Gelatin-Filled Implant Models

Silicone shells were prepared from two-component molding silicone, Best Mold PL 15, with a Pt catalyst (SP Polymer, Russia) cured at room temperature. The components were mixed in a 1:1 volume ratio and poured into polytetrafluoroethylene cylindrical custom molds with a diameter of 12 and a length of 25 mm. Ln_2_O_3_ (Ln = La, Gd, and Yb) was added to the liquid silicone with a Ln content of 20 mg·mL^−1^ in the silicone where required. In some cases, hard gelatin capsules of ~1 mL were used to fix the inner space. The shells were solidified for at least 24 h. Gelatin fillers were prepared by mixing 20 mg·mL^−1^ of La_2_O_3_ with 5 wt.% gelatin. Gelatin solutions were poured into the prepared silicone shells, refrigerated to ensure gelling, and then sealed with silicone or Ln_2_O_3_-doped silicone sheets.

**Figure 2:**
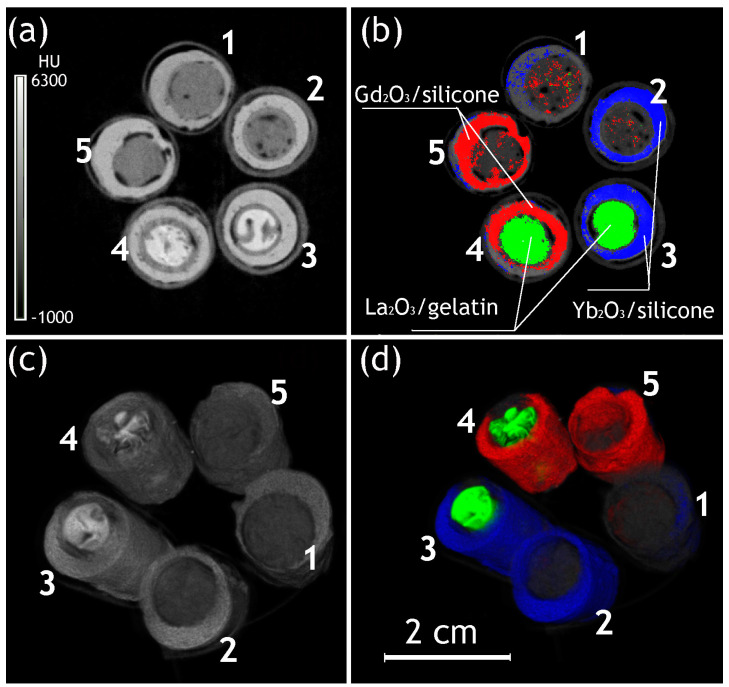
Survey scans, including transversal slices (**a**,**b**) and 3D reconstructions (**c**,**d**) of the gelatin-filled implants in energy-integrative mode (energy window 37–48 keV, (**a**,**c**)) and material determination mode (**b**,**d**). Gd—red; La—Green; Yb—yellow; 1—gelatin@silicone; 2—gelatin@(Yb_2_O_3_/silicon); 3—(La_2_O_3_/gelatin)@(Yb_2_O_3_/silicon); 4—(La_2_O_3_/gelatin)@(Gd_2_O_3_/silicon); 5—gelatin@(Gd_2_O_3_/silicon).

The phantom PCCT studies were performed in two steps. First, the survey scans including the phantom with all implants were performed to confirm the possibility of simultaneous determination of the composition and visualization of the components of the multiple implants (Table 1 and Figure 2). Second, the chosen implants were scanned separately and reconstructed with a higher resolution. The resulting images were assessed in a 37–48 keV energy window (used as an illustration of an energy-integrative scan because this energy range prevails in the emission spectra of the X-ray source) and with qualitative material determination.

The images of the 3D reconstruction of gelatin–silicone models are presented in Figure 2, and the CT numbers of the materials are summarized in Table 1. A review of the energy-integrative images produced without material determination confirmed the visualization and, to a certain extent, separation of the implant components, including the silicone shells, inner material (including heterogeneities potentially caused by gelatin shrinkage during gelling), and partially gelatin capsules, where they remained stable (Figure 2a,c). La-contrasted gelatin had a higher radiodensity than raw gelatin, but the different types of silicone shells could not be distinguished from each other.

The same images produced with the determination of materials provided higher visibility of all components, with a clear delineation of the shells from the inner materials (Figure 2b,d).

**Figure 3:**
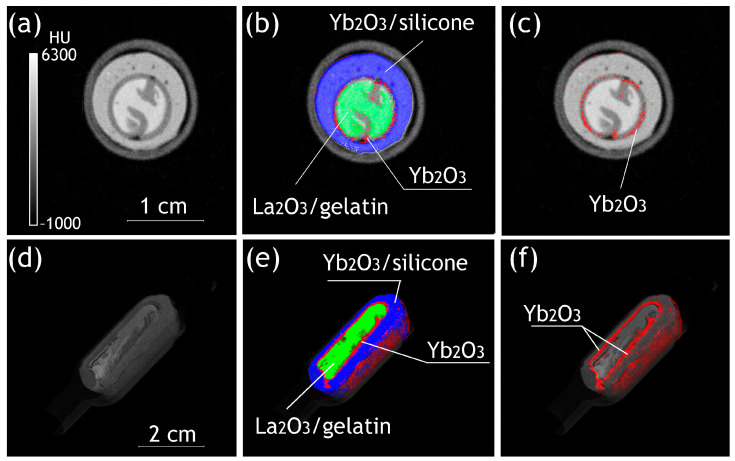
The energy-integrative (**a**,**d**) and material determination (**b**,**c**,**e**,**f**) reconstructions ((**a**–**c**)—transversal slices; (**d**–**f**)—3D visualizations) of the La_2_O_3_ + gelatin/Yb_2_O_3_ + silicone implant. Yb_2_O_3_ absorbed at the interface is shown in red.

Despite the relatively positive results of the survey scans, the subsequent scans of individual gelatin–silicone implants revealed substantial abnormalities in contrast distribution in their components. The material determination scan of the implant with an Yb-contrasted silicone shell (Figure 3) revealed the presence of small particles of Yb_2_O_3_ not bound with the silicone at both the inner and outer sides of the shell (Figure 3b,c,e,f), potentially appearing as a result of the precipitation of Yb_2_O_3_ particles at the wall of the mold during the solidification of the silicone and indicating a risk to material stability and toxicity in the case of implementation. This approach to contrast enhancement was considered sub-optimal.

### 2.3. Synthesis and Characterization of Cross-Linked Ln_2_O_3_/PAM Samples

Following the study of gelatin-filled implants, the possibility of contrasting PAM was assessed. The introduction of Ln_2_O_3_ particles into the PAM matrix without chemical modification was not successful due to heavy oxides flaking from the reaction mixture (Figure S1, Supplementary Materials). It was concluded that the size of Ln_2_O_3_ particles should be reduced and that these particles should be subsequently coated by carbon shells forming a core–shell structure with functional surface reactive groups to ensure the covalent bonding and uniform distribution of Ln_2_O_3_ in the PAM matrix.

The concept of using carbon shells for a better distribution of Ni and Fe particles in PAM was previously presented in [47]. Hydrogels based on PAM with Ag@carbon nanotubes were described in [48]. Composites of fullerene [49], carbon dots [50], multi-walled carbon nanotubes [51,52], graphene oxide [53,54], and graphite [55] with PAM, including cross-linked carbon/PAM samples [56], have also been described. However, considering the physico-chemical properties of Ln_2_O_3_, we proposed functionalizing the carbon shell in Ln_2_O_3_@C core–shell nanoparticles with allylic groups. These groups containing the C=C bond react with acrylic acid and acrylamide via the radical polymerization process with the formation of covalent bonds.

**Figure 4:**
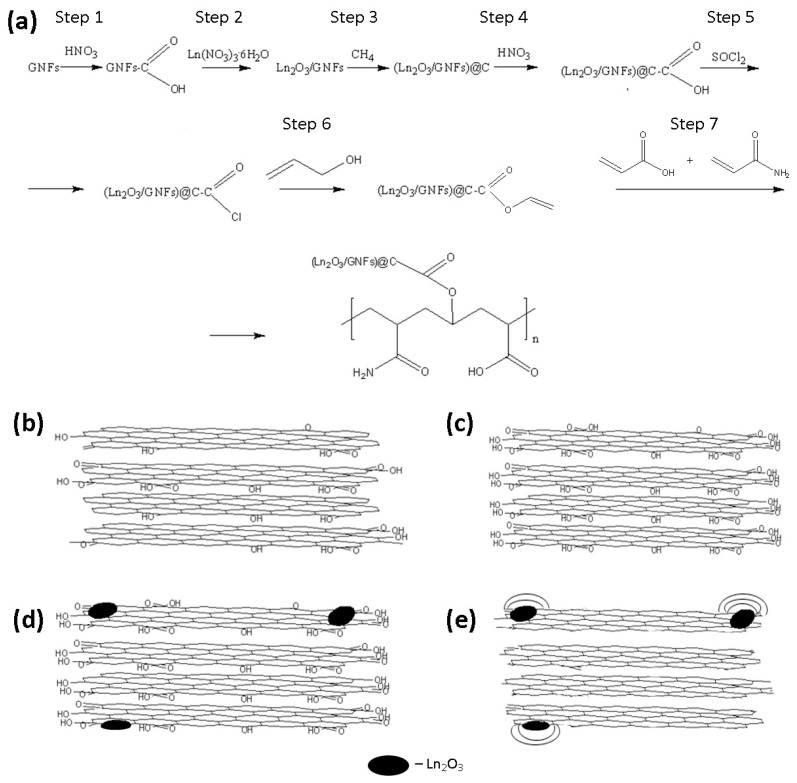
The general scheme of the synthetic route (**a**). The models of GNFs (**b**), GNFs_ox (**c**), Ln_2_O_3_/GNFs (**d**), and (Ln_2_O_3_/GNFs)@C (**e**).

**Figure 5:**
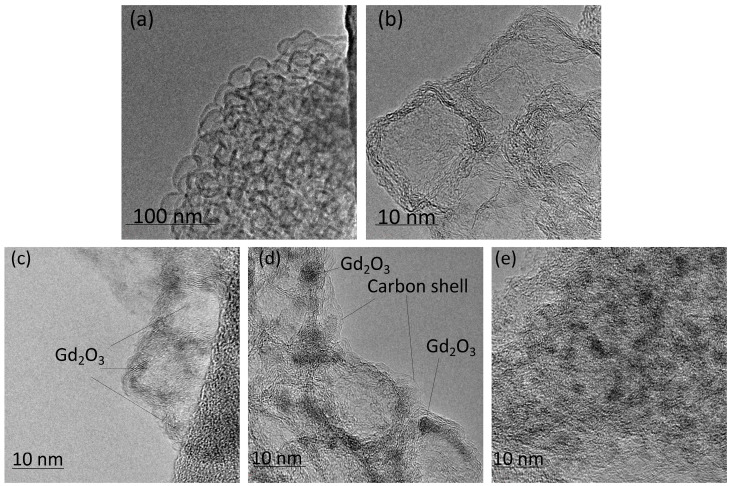
TEM images of GNFs (**a**,**b**), Gd_2_O_3_/GNFs (**c**), (Gd_2_O_3_/GNFs)@C (**d**), and (Gd_2_O_3_/GNFs)@C_ox (**e**).

The general scheme, including the seven steps of the synthetic route, is presented in Figure 4. Nanosized Ln_2_O_3_ particles could be produced by stabilization using graphene nanoflakes (GNFs). GNFs are graphitic-like flat particles with a size of ~15 × 15 nm (Figure 5a) consisting of 7–10 stacked graphene layers (Figure 5b).

Composite Ln_2_O_3_/GNFs could be obtained through the impregnation of GNFs with a Ln(NO_3_)_3_·6H_2_O solution and the further decomposition of nitrate under an inert atmosphere, as described in our previous works [23,25]. The sizes of Ln_2_O_3_ particles synthesized through the above-mentioned route were 1–2 nm according to transmission electron microscopy (TEM) (Figure 5c). Subsequent graphitization by the pyrolytic decomposition of CH_4_ produced (Ln_2_O_3_/GNFs)@C core–shell particles, which was also confirmed by TEM (Figure 5d). The surface-oxidized (Ln_2_O_3_/GNFs)@C_ox samples were produced by (Ln_2_O_3_/GNFs)@C oxidation with HNO_3_ vapors. The core–shell structure and the size of the Ln_2_O_3_ core in the (Ln_2_O_3_/GNFs)@C_ox particles were preserved (Figure 5e). It should be noted that the GNF structure was saved after all the processes and surface reactions. The structure of GNF particles is clearly demonstrated in Figure S2 in the Supplementary Materials. The composition and physico-chemical properties of Ln_2_O_3_/GNFs, (Ln_2_O_3_/GNFs)@C, and (Ln_2_O_3_/GNFs)@C_ox (Ln = La and Gd), based on X-ray photoelectron spectroscopy (XPS), electron paramagnetic resonance (EPR), Raman spectra, TEM, and thermogravimetry/differential scanning calorimetry (TG/DSC) data, are partially reported in [23,57,58,59].

**Figure 6:**
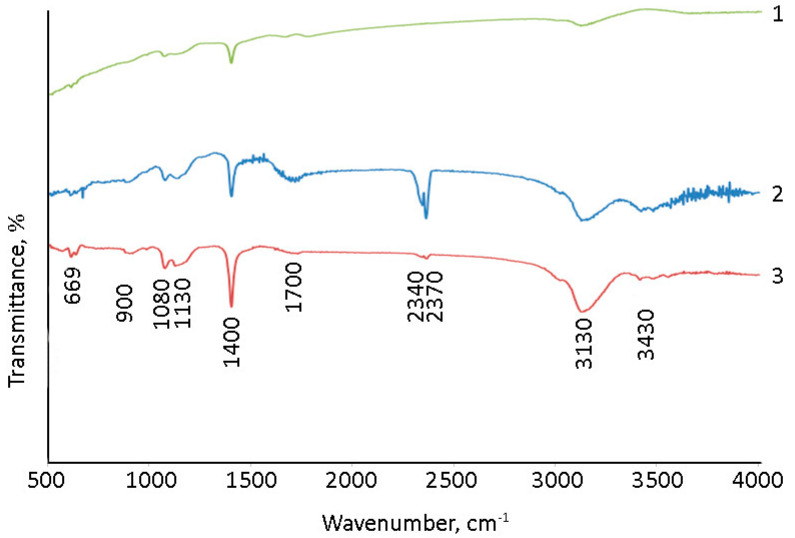
IR spectra of 1—GNFs_ox; 2—(Gd_2_O_3_/GNFs)@C_ox; and 3—(Gd_2_O_3_/GNFs)@C-COOCH_2_C_2_H_3_.

The surface of (Gd_2_O_3_/GNFs)@C_ox was further modified by reacting first with SOCl_2_ to produce an acyl chloride, and then with sodium alloxide to produce an ester. The synthesis of a nanodiamond and CNTs with surface acyl chloride groups was previously described in [60,61]. The product of the reaction of (Gd_2_O_3_/GNFs)@C-COCl with sodium alloxide was investigated by IR spectra (Figure 6). The spectra of (Gd_2_O_3_/GNFs)@C-COOCH_2_C_2_H_3_ were comparable with those of (Gd_2_O_3_/GNFs)@C_ox (Figure 6). The observed spectra were in good agreement with the spectral data for different oxidized carbon nanomaterials, such as graphene oxide [62,63], oxidized carbon nanotubes [64], and oxidized GNFs specifically (Figure 6). A wide line at 1710 cm^−1^ corresponds to the valent vibrations of the C=O fragment in the carboxyl groups. The lines at 1080 and 3130 cm^−1^ correspond to C-OH bonds in both carboxylic and hydroxyl groups. The lines at ~500–550 cm^−1^ should be associated with the Gd-O bond [65,66].

**Figure 7:**
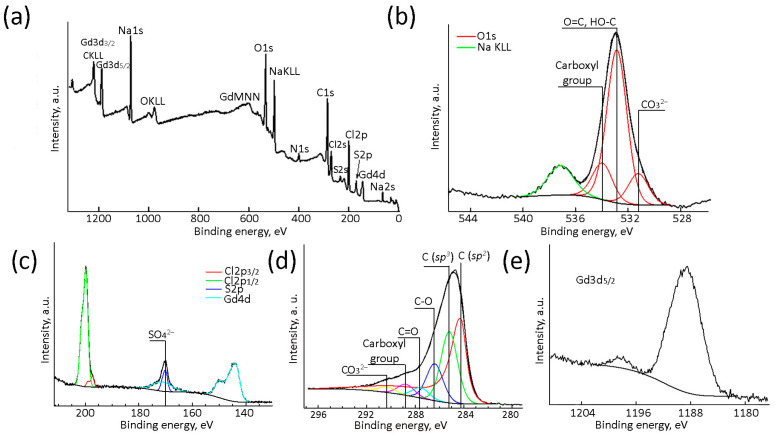
Survey (**a**), O1s (**b**), S2p (**c**), C1s (**d**), and Gd3p (**c**,**e**) high-resolution XPS spectra of (Gd_2_O_3_/GNFs)@C-COOCH_2_C_2_H_3_.

The chemical composition and the nature of atoms in the (Gd_2_O_3_/GNFs)@C-COOCH_2_C_2_H_3_ composition were confirmed by XPS (Figure 7). The sample consisted of 57.4 at.% C, 16.1 at.% O, 3.5 at.% Gd, 7.4 at.% Na, 9.8 at.% Cl, 3.9 at.% N, and 1.8 at.% S (Figure 7a). The sample contained NaCl (Figure 7b,c) and Na_2_SO_4_ (Figure 7c) as by-products of a metathesis reaction between (Gd_2_O_3_/GNFs)@C-COCl and NaOCH_2_C_2_H_3_ according to step 6 and the formation of acyl chloride via step 5 (Figure 4). Nitrogen atoms were predominantly found in the form of oxides (Figure S3, Supplementary Materials) and remained in the product after the surface oxidation phase [57,59]. High-resolution deconvoluted C1s XPS spectra of (Gd_2_O_3_/GNFs)@C-COOCH_2_C_2_H_3_ (Figure 7d) contained different carbon species with binding energies of 284.4, 285.2, 286.4, 287.6, 288.8, and 290.3 eV corresponding to *sp^2^*- and *sp^3^*-hybridized C, C–O, ketone C=O, and hydroxyl C(O)O groups and CO_3_^2−^. This confirmed the presence of all possible carbon groups expected in (Gd_2_O_3_/GNFs)@C-COOCH_2_C_2_H_3_. The CO_3_^2−^ group in the spectra was presumably related to Gd carbonate (Figure 7e) [67] which was formed as the primary Gd phase [23]. This assumption is supported by O1s XPS spectra showing binding energies of 531.2, 532.9, and 534.0 eV corresponding to CO_3_^2−^, C=O/C-OH, and COO- groups (Figure 7b).

**Figure 8:**
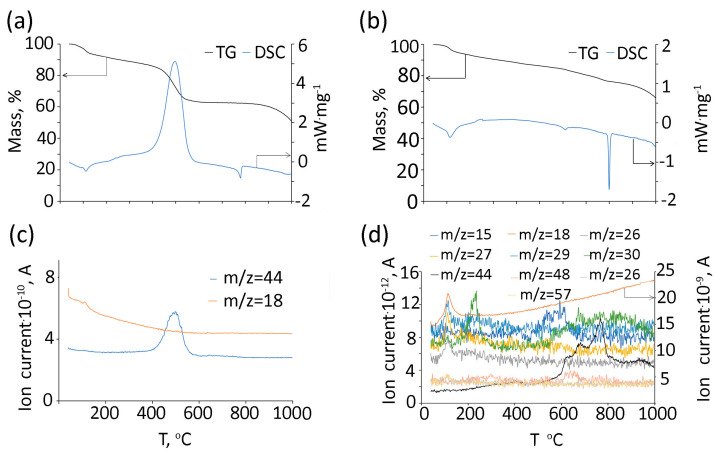
TG and DSC curves (**a**,**b**) and gas products (**c**,**d**) of (Gd_2_O_3_/GNFs)@C-COOCH_2_C_2_H_3_ under air (**a**,**c**) and Ar (**b**,**d**) atmospheres.

TG/DSC data of (Gd_2_O_3_/GNFs)@C-COOCH_2_C_2_H_3_ with mass-spectra of evolved gasses for the measurements in the air and Ar atmosphere are depicted in Figure 8a,c and Figure 8b,d, respectively. During heating in an oxidizing atmosphere, adsorption water is released with heat absorption (Figure 8a,c). Then, the carbon shell burns with an intense release of heat and CO_2_ peaking at approximately 500 °C. This is followed by an endothermic peak caused by the melting of NaCl, a by-product of the synthesis process. At temperatures above 800 °C, the salt decomposes, releasing chlorine. The residual mass of 50% corresponds to Gd_2_O_3_. In an inert atmosphere, the composition of gaseous products is more complex (Figure 8b,d). Water (*m*/*z* = 18, 17, and 16) and carbon dioxide (*m*/*z* = 44, 12, and 45) are released in a wide temperature range. The peak of water release is observed at a temperature slightly above 110 °C. There are also peaks for *m*/*z* = 29 (C_2_H_5_^+^), 27 (C_2_H_3_^+^), and 26 (C_2_H_2_^+^). Amidst background noise, peaks are also observed for *m*/*z* = 57(C_3_H_5_O^+^) and 56 (C_3_H_4_O^+^). Above 130 °C, the *m*/*z* = 30 peak begins to appear, reaching its maximum at around 230 °C. At approximately 590 °C, the maximum ion current for *m*/*z* = 15 (CH_3_^+^) is observed. Between 600 and 700 °C, SO_2_ is released (*m*/*z* = 64, 48) due to the by-product of synthesis in accordance with XPS data. The maximum CO_2_ emission is observed near 770 °C, but it is released over a much broader temperature range compared to an oxidizing atmosphere (Figure 8d).

**Figure 9:**
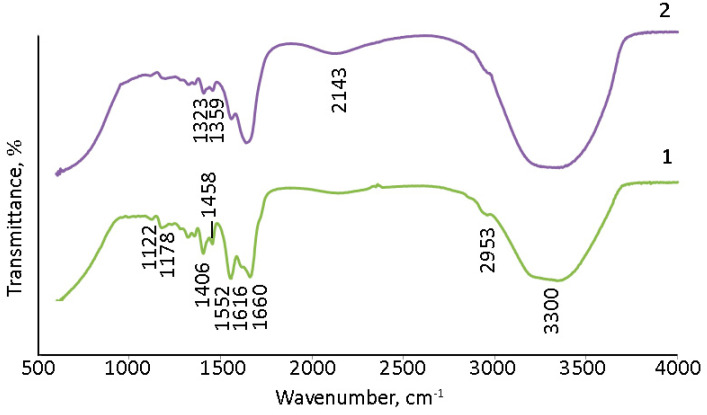
IR spectra: **1**—PAM; **2**—(Gd_2_O_3_/GNFs)@C-COOCH_2_C_2_H_3_/PAM.

The surface modification of (Gd_2_O_3_/GNFs)@C particles by allylic groups improves their degree of dispersion in solvents [60], and they serve as functional groups reacting with the monomers acrylic acid and acrylamide via radical mechanism polymerization [60]. The IR spectra of PAM and (Gd_2_O_3_/GNFs)@C-COOCH_2_C_2_H_3_/PAM are presented in Figure 9. The spectra of PAM and PAM with cross-linked Gd_2_O_3_, i.e., (Gd_2_O_3_/GNFs)@C-COOCH_2_C_2_H_3_ particles, are similar. The interpretation of the IR spectra was based on [68,69]. The wide lines at 3300 cm^−1^ correspond to H_2_O and amide NH_2_ asymmetric stretching groups. The lines at 2953 and 1406 cm^−1^ are associated with C-H asymmetric and symmetric stretching, respectively. The lines at 1660 and 1616 cm^−1^ correspond to the stretching of primary and secondary amide C=O groups. C–O–C groups could contribute to the lines at 1178 and 1178 cm^−1^. The lines at ~1350 cm^−1^ could be interpreted as unreacted C=C groups and C–O–H in plane bending because of their comparable wavenumbers [68].

PAM has a unique property, namely the ability to adsorb water with swelling [70,71]. The swelling of PAM and the cross-linked Ln_2_O_3_/PAM composite was evaluated according to the increase in the weight of samples after water sorption. Two hundred milliliters of distilled water was added to a solid weighed portion of 0.50 g of the polymer sample and kept for 24 h, after which it was filtered through a sieve with a mesh size of 0.5 mm, and the weight of the swollen residue was determined. The swelling values of PAM and Gd_2_O_3_/PAM were 420 and 320 g·g^−1^. The densities of PAM and the (Ln_2_O_3_/GNFs)@C-COOCH_2_C_2_H_3_/PAM (Ln = La, Gd) composites determined as the ratio between their mass and volume were 1.27, 1.04, and 1.20 g·mL^−1^, accordingly. The qualitative determination of Gd in the water solution demonstrated the absence of Gd, which indirectly indicates the binding of Gd_2_O_3_ to the polymer matrix.

**Figure 10:**
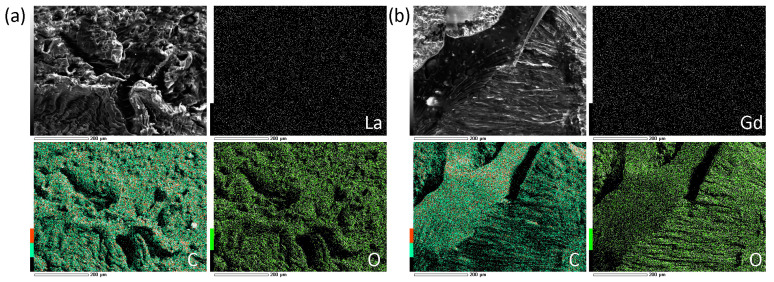
SEM images and surface Ln, C, and O mapping of (La_2_O_3_/GNFs)@C-COOCH_2_C_2_H_3_/PAM (**a**) and (Gd_2_O_3_/GNFs)@C-COOCH_2_C_2_H_3_/PAM (**b**).

The uniformity of Ln_2_O_3_ distribution in the PAM matrix was confirmed by scanning electron microscopy (SEM) images and surface element mapping (Figure 10). The contents of La and Gd were 0.61 and 0.30 wt.% according to EDX data, which are in good agreement with the synthesis process.

### 2.4. Preparation and Phantom PCCT Studies of PAM-Filled Implant Models

PAM implant models were produced from standard silicone tubing filled by (Ln_2_O_3_/GNFs)@C-COOCH_2_C_2_H_3_/PAM (Ln = La or Gd) or PAM and sealed using silicone. The implants were solidified for 24 h before studying them. The PCCT studies were performed in one step (without survey scans) due to a smaller number of implants requiring assessment. The energy-integrative reconstructions were produced from a separate scan with a single energy threshold of 30 keV.

**Figure 11:**
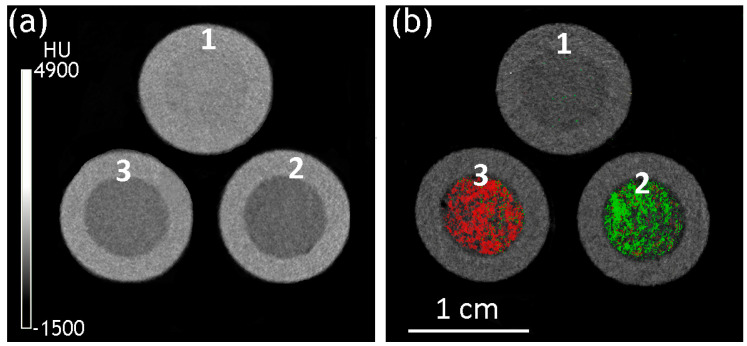
Transversal slices of the reconstructed PAM-filled implants in energy-integrative (**a**) and material determination (**b**) modes: **1**—PAM/silicone; **2**—(La_2_O_3_/GNFs)@C-COOCH_2_C_2_H_3_/PAM/silicone; and **3**—(Gd_2_O_3_/GNFs)@C-COOCH_2_C_2_H_3_/PAM/silicone. Green—La; red—Gd.

The transversal slices of the reconstructed implant models with a silicone shell and (Ln_2_O_3_/GNFs)@C-COOCH_2_C_2_H_3_/PAM Ln = La (a) and Gd (b) filler are presented in Figure 11. In both cases, the fillers and shells could be visualized. No visible abnormalities were detected in the distribution of the CA. The average X-ray attenuation coefficient (Table 2) of both (Ln_2_O_3_/GNFs)@C-COOCH_2_C_2_H_3_/PAM fillers was lower compared to raw PAM, which could be explained by the presence of low-density GNFs compensating for the higher attenuation of the Ln component of the material. However, the contrasted fillers had characteristic attenuation spectra, making it possible to separate them from the silicone shells and from each other.

The visualization and successful Ln determination in PAM fillings were performed with a Ln concentration in (Ln_2_O_3_/GNFs)@C-COOCH_2_C_2_H_3_/PAM of 4.5 mg·mL^−1^. In our past works, we demonstrated that the lower concentration threshold for Ln determination by PCCT was ~2.5 mg·mL^−1^ [23], while further optimization of material determination algorithms allows for it to be reduced to under ~1 mg·mL^−1^ [44]. This confirms the higher potential of Ln contrast enhancement compared to other approaches, such as iodine-based contrasts [72], requiring higher CA concentrations (>4 mg·mL^−1^ iodine in clinical applications) and not allowing the simplified production of multiple CAs for simultaneous visualization. This also confirms the high potential of the proposed materials from a biocompatibility perspective. Their core–shell structure provides additional shielding for Ln nanoparticles, which is expected to decrease their cytotoxicity compared to simpler nanoparticles with direct contact between Ln and biological media [73]. However, the biocompatibility of the contrasted materials requires further comprehensive studies due to the different nature of possible exposure compared to the commonly studied contrasts for short-term IV administration.

## 3. Conclusions

The results of this study demonstrate that Ln_2_O_3_ contrasting can be an effective method for the visualization of hydrogel-based implants for diagnostic studies involving CT or PCCT. Various combinations of Ln can be used, although the suitability and effectiveness of each individual combination should be confirmed through calibration experiments. The inclusion of CA particles into the components of the implant at the stage of polymerization without covalent bonding may give sub-optimal results due to the redistribution of the contrast, including the dissemination of the contrast at the surface of the implant. Covalent bonding of the CA into the hydrogel structure with the formation of the new cross-linked Ln_2_O_3_/PAM composite increases the uniformity of its distribution in the material and increases its stability. Ln_2_O_3_/PAM composites can be visualized with specific Ln determination in implantable systems with Ln concentrations below 5 mg·mL^−1^, demonstrating their high potential compared to other contrast enhancement approaches. The structure of the proposed materials allows for low cytotoxicity to be anticipated, although this requires further comprehensive studies due to their different uses and possible mechanisms of exposure compared to the Ln-based nanoparticle contrasts described in the literature.

## 4. Materials and Methods

### 4.1. Synthesis of Cross-Linked Ln_2_O_3_/PAM Samples

The general scheme of the synthetic route, including seven steps, and models of component structures are shown in Figure 3. The synthesis and product characterization of steps 1–4 are described in detail in our previous works [57,58,59]. Steps 5–7 were originally described for modifying the nanodiamond surface in [60] and customized for the composites produced in step 4.

#### 4.1.1. Synthesis of Contrast Agents

In brief, GNFs were synthesized by the pyrolysis of hexane (99.8%, Reachim) at 900 °C in the presence of a MgO template according to [74]. MgO was removed by boiling in HCl (99.9%, Reachim). The obtained GNFs were washed with distilled water and dried at 110 °C.

Step 1. GNFs were oxidized by HNO_3_ (Component-Reaktiv) refluxing for 1 h to produce a GNFs_ox sample. The content of oxygen on the GNFs_ox surface was 11.1 at.% [59].

Step 2. Then, the GNFs_ox samples were impregnated with Ln(NO_3_)_3_·6H_2_O (Ln = La and Gd) (99%, China Northern Rare Earth Group High-Tech Co., Ltd., Baotou, China) ethanolic solution. The solvent was evaporated slowly using an ultrasound bath. After that, the solid sediment was heated at 400 °C with nitrogen (99.999%, NII KM, Moscow, Russia) flow for 45 min to decompose nitrate and obtain a Ln_2_O_3_/GNF composite [23].

Step 3. The core–shell structure (Ln_2_O_3_/GNFs)@C was achieved by methane decomposition over Ln_2_O_3_/GNFs particles [58]. The Ln_2_O_3_/GNFs sample was placed into the fixed-bed reactor, purged with nitrogen, and heated to 450 °C. After that, methane (99.99% Moscow Gas Processing Plant, Moscow, Russia) was passed through the reaction mixture for 15–20 min. The product (Ln_2_O_3_/GNFs)@C was used without purification.

Step 4. The surface oxidation of (Ln_2_O_3_/GNFs)@C particles was performed via HNO_3_ gas phase treatment according to recommendations [57,59]. The (Ln_2_O_3_/GNFs)@C sample was put into the open glass vessel and treated with HNO_3_ vapors for 1 h. Adsorbed HNO_3_ was washed by dt. H_2_O, and the resulting product (Ln_2_O_3_/GNFs)@C_ox was dried at 110 °C.

Step 5. The next surface modification process for (Ln_2_O_3_/GNFs)@C_ox involved the substitution of the hydroxyl group in a carboxylic group with a chlorine atom to produce acyl chloride. For this purpose, 1.0 g of (Ln_2_O_3_/GNFs)@C_ox was placed in a round-bottomed double-crested bowl equipped with a thermometer and a reflux condenser. Fifty milliliters of SOCl_2_ was added. The reaction mixture was refluxed at 80 °C for 24 h. The unreacted SOCl_2_ was evaporated to produce acyl chloride (Ln_2_O_3_/GNFs)@C-COCl.

Step 6. The synthesis of the ester was carried out through the reaction of acyl chloride (Ln_2_O_3_/GNFs)@C-COCl with sodium alloxide. Metallic Na in the amount of 0.46 g was added to 50 mL of allylic alcohol (>99%, Himkraft Ltd., Kaliningrad, Russia). After the reaction, the NaOCH_2_C_2_H_3_ solution was dropped to (Ln_2_O_3_/GNFs)@C-COCl under an Ar (99.993%, Logica Ltd., Moscow, Russia) atmosphere. The reaction mixture was refluxed for 8 h; the solid product (Ln_2_O_3_/GNFs)@C-COOCH_2_C_2_H_3_ was centrifugated at 6000 rpm and washed with ethylacetate two times. The product was kept at 0 °C in the dark until the next stage.

#### 4.1.2. Synthesis of PAM

PAM was synthesized according to the standard procedure of the copolymerization of potassium acrylate, acrylamide, and the crosslinking agent N,N′-methylenebisacrylamide with (NH_4_)_2_S_2_O_8_ as a catalyst of the radical polymerization process [75,76,77]. Acrylic acid (99.98%, Vekton, St. Petersburg, Russia) in the amount of 7.1 g was dissolved in 20 mL of water and neutralized to pH 7 with 50 mL of a KOH solution. Acrylamide (99.9%, Vekton, St. Petersburg, Russia) in the amount of 9.12 g was dissolved in 40 mL of H_2_O. Both solutions were combined, and then 0.0270 g of N,N′-methylenebisacrylamide (99.99%, Amresco LLC, Boise, ID, USA) was added. The mixture was blown by Ar. Solid (NH_4_)_2_S_2_O_8_ (99%, Ruschem, Moscow, Russia) was added. The reaction mixture was heated to 50 °C to trigger the gelling reaction and then self-heated to 90 °C. The product was washed with water and dried at 130 °C for 10 h.

#### 4.1.3. Synthesis of Ln_2_O_3_/PAM Composites

The composites of PAM with Ln_2_O_3_ (Ln = La, Gd, and Yb) (99.99%, China Northern Rare Earth Group High-Tech Co., Ltd., Baotou, China) were synthesized by sewing the suspended composites in the PAM matrix via the copolymerization of monomers according to [78]. Solid Ln_2_O_3_ was added to the water solution of potassium acrylate and acrylamide (the concentration of monomers was in accordance with the previous synthesis description) with vigorous stirring. The mass content of Ln_2_O_3_ varied from 5 to 25 wt.%. N_2_ was blown out, and a water solution of N,N′-methylenebisacrylamide was titrated to the reaction mixture. The polymerization reaction started after Na_2_S_2_O_8_ addition. However, in all cases, Ln_2_O_3_ flaked out.

Step 7. A cross-linked sample, (Gd_2_O_3_/GNFs)@C-COOCH_2_C_2_H_3_/PAM, was obtained by adding solid (Gd_2_O_3_/GNFs)@C-COOCH_2_C_2_H_3_ powder into the reaction mixture of potassium acrylate, acrylamide, and N,N′-methylenebisacrylamide before the copolymerization process. The mixture was intensively stirred and purged with Ar for 20 min. (NH_4_)_2_S_2_O_8_ was added as an initial agent. The reaction was generally similar to the synthesis of polyacrylamide. The contents of Ln in the composition of (Ln_2_O_3_/GNFs)@C-COOCH_2_C_2_H_3_/PAM were 0.39 and 0.30 wt.% for the La and Gd composites, respectively.

### 4.2. Methods

The thermal stability of (Gd_2_O_3_/GNFs)@C-COOCH_2_C_2_H_3_ was studied using a simultaneous thermal analyzer, STA 449 C Jupiter, combined with a quadrupole mass-spectrometer, QMS 403 C Aeolos (both NETZSCH, Selb, Germany). The material in the Al_2_O_3_ crucible was heated at a rate of 10 °C·min^−1^ from 40 to 1000 °C, both in oxidizing (airflow of 80 mL·min^−1^) and inert (Ar flow of 80 mL·min^−1^) atmospheres. The weighing block was purged with a flow of argon protective gas of 40 mL·min^−1^. The signals of thermogravimetry (TG), differential thermogravimetry (DTG), differential scanning calorimetry (DSC), and ion currents corresponding to certain *m*/*z* ratios of evolved gasses were recorded during the heating process. A baseline correction was applied for the TG/DSC curves using the measurement with an empty crucible.

The morphology and particle size were studied by SEM using JEOL JSM-6390LA and JEOL JCM-6000 instruments (JEOL Ltd., Tokyo, Japan). TEM images were obtained using JEOL 2100 F/Cs (Jeol, Tokyo, Japan) operating at 200 kV and equipped with a UHR pole tip as well as a spherical aberration corrector (CEOS, Heidelberg, Germany) and EEL spectrometer (Gatan, Munich, Germany).

The materials were studied using an FTIR spectrometer, FSM 1202 (Infraspek, Moscow, Russia). Transmission spectra were obtained by accumulating 30 scans with a resolution of 4 cm^−1^. Powdered samples were pressed into tablets with KBr (98.5%, BLD Pharmatech Ltd. China, Shanghai, China) using a hydraulic press (OMEC, Brescia, Italy). A reference spectrum was registered from a pure KBr pellet. Polyacrylamide samples were pressed to a ZnSe crystal of the multi-reflection ATR accessory.

The XPS spectra were recorded on an Axis Ultra DLD spectrometer (Kratos Analytical, Manchester, UK) using monochromatic AlKα radiation (1486.7 eV). The pass energies of the analyzer were 160 eV for survey spectra and 40 eV for high-resolution scans. The relative error for the element concentrations was about 5%.

### 4.3. PCCT Studies

PCCT studies were conducted using a MARS Bioimaging microtomograph (MARS Bioimaging Ltd., Christchurch, New Zealand) equipped with 2 Medipix 3RX CZT pixel detectors [79] with a sensor size of 14 mm × 14 mm and a thickness of 1 mm. The material determination scans of the gelatin-filled implant prototypes were performed with energy thresholds of 7, 25, 37, 48, 58, 72, 80, and 90 keV, which were chosen based on the K-edge energies of the studied Ln (38.9, 50.2, and 61.3 keV for La, Gd, and Yb, respectively). The scans of the PAM-filled implant prototypes were performed with energy thresholds of 7, 25, 36, 48, and 60 keV since only La and Gd were used. The images illustrating the energy-integrative reconstructions were produced using a 37–48 keV energy window (for gelatin-filled implant prototypes) or by separate scans within a single energy threshold at 30 keV (for PAM-filled implant prototypes). The scans were performed in continuous circular mode with automated flat field correction, a geometric magnification of 1.7, and rotation steps of 1° in survey and calibration scans and 0.5° in the scans of individual implants. The reconstruction was performed with a voxel size of 0.12 mm for survey scans and 0.06 mm for calibration scans and scans of individual implants. Because of the detector size, the total scanning time in all studies was approximately 70 min, including 55 min for acquiring the actual projections and 15 min for acquiring the flat field data for all camera positions (up to 4 for each position of the sample). Scanning was performed with temperature conditioning, keeping the detector temperature under 16 °C. Visualization was performed using the Mars Vision 2.0 software (MARS Bioimaging Ltd., Christchurch, New Zealand). The energy-integrative attenuation data were additionally translated into the Hounsfield scale using additional water samples scanned with the same parameters. The CT numbers (HU values) were measured from transversal slices for the area of at least 2 mm^2^ and presented as mean ± SD.

## Figures and Tables

**Table 1:** CT numbers of materials used in gelatin-filled implants (mean ± SD).

Shell	CT Number (HU)	Filling	CT Number (HU)
Silicone	649 ± 43	Gelatin	75 ± 5
Gd_2_O_3_-doped silicone	1041 ± 69	Gelatin capsules	356 ± 28
Yb_2_O_3_-doped silicone	1056 ± 71	La_2_O_3_-doped gelatin	2211 ± 119

**Table 2:** The CT numbers of the materials used in PAM-filled implants (mean ± SD).

Shell	CT Number (HU)	Filling	CT Number (HU)
Silicone	1025 ± 84.6	PAM	1104 ± 985
		(La_2_O_3_/GNFs)@C-COOCH_2_C_2_H_3_/PAM	264 ± 25
		Gd_2_O_3_/GNFs)@C-COOCH_2_C_2_H_3_/PAM	171 ± 15

## Data Availability

The original contributions presented in this study are included in the article/supplementary material. Further inquiries can be directed to the corresponding authors.

## Supplementary Materials

The following supporting information can be downloaded at https://www.mdpi.com/article/10.3390/gels10120807/s1, Figure S1: SEM images of PAM (a) and mixture of La_2_O_3_ (b) and Gd_2_O_3_ (c) with PAM synthesized during polymerization of acrylic acid and acrylamide in presence of Ln_2_O_3_. Figure S2: HRTEM (a,b) and HAADF-STEM (c) images with EELS spectra of (Gd_2_O_3_/GNFs)@C-COOCH_2_C_2_H_3_ (d). Figure S3: N1s XPS spectra of (Gd_2_O_3_/GNFs)@C-COOCH_2_C_2_H_3_.

## References

[1] Chamkouri H., Chamkouri M. (2021). A Review of Hydrogels, Their Properties and Applications in Medicine. Am. J. Biomed. Sci. Res..

[2] Peppas N.A., Hilt J.Z., Khademhosseini A., Langer R. (2006). Hydrogels in Biology and Medicine: From Molecular Principles to Bionanotechnology. Adv. Mat..

[3] Hameed H., Faheem S., Paiva-Santos A.C., Sarwar H.S., Jamshaid M. (2024). A Comprehensive Review of Hydrogel-Based Drug Delivery Systems: Classification, Properties, Recent Trends, and Applications. AAPS PharmSciTech.

[4] Martinea G., Begines B., Pajuelo E., Vazquez J., Rodriguez-Alberlo L.M., Cofini D., Torres Y., Alcudia A. (2023). Versatile Biodegradable Poly(acrylic acid)-Based Hydrogels Infiltrated in Porous Titanium Implants to Improve the Biofunctional Performance. Biomacromolecules.

[5] Wong T., Lo L.W., Fung P.Y.E., Lai H.Y.M., She H.L.H., Ng W.K.C., Kwok K.M.K., Lee C.M. (2016). Magnetic resonance imaging of breast augmentation: A pictorial review. Insights Imaging.

[6] Dong Y.C., Bouché M., Uman S., Burdick J.A., Cormode D.P. (2021). Cormode Detecting and Monitoring Hydrogels with Medical Imaging. ACS Biomater. Sci. Eng..

[7] Wu H.-H., Weng Y.-T., Chou Y.-Y., Wang C.-H. (2023). Rupture of 40-year-old silicone gel breast implants: A case report. BMC Geriatr..

[8] Mottu F., Rüfenacht D.A., Doelker E. (1999). Radiopaque polymeric materials for medical applications. Current aspects of biomaterial research. Investig. Radiol..

[9] Li Q.F., Wang J.T., Wang Z. Implementation of functional integration of hydrogel matrices with rare earth elements and related applications.

[10] Koc M.M., Aslan N., Kao A.P., Barber A.H. (2019). Evaluation of X-Ray tomography contrast agents: A review of production, protocols, and biological applications. Microsc. Res. Technol..

[11] Becerra C.F., Silva V.B., Ahmed E., Bear J.C., Feng Z., Chau D.Y.S., Parker S.G., Halligan S., Lythgoe M.F., Stuckey D.J. (2024). X-Ray Visible Protein Scaffolds by Bulk Iodination. Adv. Sci..

[12] Houston K.R., Brosnan S.M., Burk L.M., Lee Y.Z., Luft J.C., Ashby V.S. (2017). Iodinated polyesters as a versatile platform for radiopaque biomaterials. J. Polym. Sci. A Polym. Chem..

[13] Kiran S., James N.R., Joseph R., Jayakrishnan A. (2009). Synthesis and characterization of iodinated polyurethane with inherent radiopacity. Biomaterials.

[14] Davy K.W.M., Anseau M.R. (1996). Novel iodinated methacrylates as X-Ray opaque denture base polymers. J. Mater. Sci. Lett..

[15] Barnett B.P., Arepally A., Stuber M., Arifin D.R., Kraitchman D.L., Bulte J.W. (2011). Synthesis of magnetic resonance-, X-Ray- and ultrasound-visible alginate microcapsules for immunoisolation and noninvasive imaging of cellular therapeutics. Nat. Protoc..

[16] Patrick P.S., Bear J.C., Fitzke H.E., Zaw-Thin M., Parkin I.P., Lythgoe M.F., Kalber T.L., Stuckey D.J. (2020). Radio-metal cross-linking of alginate hydrogels for non-invasive in vivo imaging. Biomaterials.

[17] Dong Y.C., Kumar A., Rosario-Berrios D.N., Si-Mohamed S., Hsu J.C., Nieves L.M., Douek P., Noёl P.B., Cormode D.P. (2022). Ytterbium Nanoparticle Contrast Agents for Conventional and Spectral Photon-Counting CT and Their Applications for Hydrogel Imaging. ACS Appl. Mater. Interfaces.

[18] Wu Y., Ye Z., Chen J., Deng L., Song B. (2023). Photon Counting CT: Technical Principles, Clinical Applications, and Future Prospects. Acad. Radiol..

[19] Willemink M.J., Persson M., Pourmorteza A., Pelc N.J., Fleischmann D. (2018). Photon-counting CT: Technical Principles and Clinical Prospects. Radiology.

[20] Douek P.C., Boccalini S., Oei E., Cormode D., Pourmorteza A., Boussel L., Si-Mohamed S., Budde R. (2023). Clinical Applications of Photon-counting CT: A Review of Pioneer Studies and a Glimpse into the Future. Radiology.

[21] Bie J., Straten M., Booij R., Bos D., Dijkshoorn M.L., Hirsch A., Sharma S.P., Oei E.H.G., Budde R.P.J. (2023). Photon-counting CT: Review of initial clinical results. Eur. J. Radiol..

[22] Lachance C., Horton J. (2024). Photon-Counting CT: High Resolution, Less Radiation: Emerging Health Technologies.

[23] Suslova E.V., Kozlov A.P., Shashurin D.A., Rozhkov V.A., Sotenskii R.V., Maximov S.V., Savilov S.V., Medvedev O.S., Chelkov G.A. (2022). New Composite Contrast Agents Based on Ln and Graphene Matrix for Multi-Energy Computed Tomography. Nanomaterials.

[24] Kim J., Bar-Ness D., Si-Mohamed S., Coulon P., Blevis I., Douek P., Cormode D.P. (2018). Assessment of candidate elements for development of spectral photon-counting CT specific contrast agents. Sci. Rep..

[25] Suslova E., Shashurin D., Kozlov A., Maximov S.V., Rozhkov V.A., Sotenskii R.V., Savilov S.V., Medvedev O.S., Chelkov G.A. (2022). Development of La-graphene composite contrasting agents for photon-counting computed tomography. Funct. Mater. Lett..

[26] Dunning C.A.S., O’connell J., Robinson S.M., Murphy K.J., Frencken A.L., van Veggel F.C.J.M., Iniewski K., Bazalova-Carter M. (2022). Multi energy Computed Tomography of Lanthanide Contrast Agents with a High-Flux 330-Mm-Pitch Cadmium Zinc Telluride Detector in a Table-Top System. J. Med. Imaging.

[27] Smith K., Getzin M., Garfield J.J., Suvarnapathaki S., Camci-Unal G., Wang G., Gkikas M. (2019). Nanophosphor-Based Contrast Agents for Spectral X-Ray Imaging. Nanomaterials.

[28] Richtsmeier D., Dunning C.A.S., Iniewski K., Bazalova-Carter M. (2020). Multi-Contrast K-Edge Imaging on a Bench-Top Multi energy CT System: Acquisition Parameter Study. J. Instrum..

[29] Ostadhossein F., Tripathi I., Benig L., LoBato D., Moghiseh M., Lowe C., Raja A., Butler A., Panta R., Anjomrouz M. (2020). Multi-“Color” Delineation of Bone Microdamages Using Ligand-Directed Sub-5 Nm Hafnia Nanodots and Photon Counting CT Imaging. Adv. Funct. Mater..

[30] Amato C., Klein L., Wehrse E., Rotkopf L.T., Sawall S., Maier J., Ziener C.H., Schlemmer H., Kachelrieß M. (2020). Potential of Contrast Agents Based on High-Z Elements for Contrast-enhanced Photon-counting Computed Tomography. Med. Phys..

[31] Cormode D.P., Roessl E., Thran A., Skajaa T., Gordon R.E., Schlomka J.-P., Fuster V., Fisher E.A., Mulder W.J.M., Proksa R. (2020). Atherosclerotic Plaque Composition: Analysis with Multicolor CT and Targeted Gold Nanoparticles. Radiology.

[32] Do C., DeAguero J., Brearley A., Trejo X., Howard T., Escobar G.P., Wagner B. (2020). Gadolinium-Based Contrast Agent Use, Their Safety, and Practice Evolution. Kidney360.

[33] Holmes B., Sanampudi S., Ananthakrishnan L. (2024). Diagnostic CT cystography with diluted gadolinium-based contrast: A viable alternative to an iodinated contrast-based cystogram. Urol. Case Rep..

[34] Yang J., Zhao Q., Zang Z., Zhang S., Wang Z., Li L., Yu X., Yang X., Lu Z., Zhang X. (2022). A dual-mode T1 MRI/CT contrast agent of Gd_2_O_3_/Au@MSNs for tumor imaging with high performance. Materialia.

[35] Levine D., McDonald R.J., Kressel H.Y. (2018). Gadolinium Retention After Contrast-Enhanced MRI. JAMA.

[36] Fatima A., Ahmad M.W., Al Saidi A.K.A., Choudhury A., Chang Y., Lee G.H. (2021). Recent Advances in Gadolinium Based Contrast Agents for Bioimaging Applications. Nanomaterials.

[37] Wang F., Peng E., Zheng B., Li S.F.Y., Xue J.M. (2015). Synthesis of Water-Dispersible Gd2O3/GO Nanocomposites with Enhanced MRI T1 Relaxivity. J. Phys. Chem. C.

[38] Pellico J., Ellis C.M., Davis J.J. (2019). Nanoparticle-Based Paramagnetic Contrast Agents for Magnetic Resonance Imaging. Contrast Media Mol. Imaging.

[39] Li Z., Guo J., Zhang M., Li G., Hao L. (2022). Gadolinium-Coated Mesoporous Silica Nanoparticle for Magnetic Resonance Imaging. Front. Chem..

[40] Miao X., Ho S.L., Tegafaw T., Cha H., Chang Y., Oh I.T., Yaseen A.M., Marasini S., Ghazanfari A., Yue H. (2018). Stable and non-toxic ultrasmall gadolinium oxide nanoparticle colloids (coating material = polyacrylic acid) as high-performance T1 magnetic resonance imaging contrast agents. RSC Adv..

[41] Davies J., Siebenhandl-Wolff P., Tranquart F., Jones P., Evans P. (2022). Gadolinium: Pharmacokinetics and toxicity in humans and laboratory animals following contrast agent administration. Arch. Toxicol..

[42] Gadavist (Gadobutrol) Injection, for Intravenous Use. Labelling Information. https://www.accessdata.fda.gov/drugsatfda_docs/label/2011/201277s000lbl.pdf.

[43] Akhtar M.J., Ahamed M., Alhadlaq H., Alrokayan S. (2019). Toxicity Mechanism of Gadolinium Oxide Nanoparticles and Gadolinium Ions in Human Breast Cancer Cells. Curr. Drug Metab..

[44] Sotenskii R.V., Rozhkov V.A., Shashurin D.A., Suslova E.V., Chelkov G.A. (2024). Novel algorithm for qualitative and quantitative material analysis by the K-edges for photon-counting computed tomography. JINST.

[45] Bateman C.J., Knight D., Brandwacht B., Mc Mahon J., Healy J., Panta R., Aamir R., Rajendran K., Moghiseh M., Ramyar M. (2018). MARS-MD: Rejection based image domain material decomposition. JINST.

[46] Suslova E., Shashurin D., Zoirova Z., Shumyantsev A., Medvedev O., Chelkov G. (2024). Gd_2_O_3_-based contrasting agents for photon-counting computed tomography: Effect of structure, composition, and particle size. Mater. Chem. Phys..

[47] Mikhnevich E.A., Safronov A.P., Beketov I.V., Medvedev A.I. (2020). Carbon coated Nickel Nanoparticles in Polyacrylamide Ferrogels: Interaction with Polymeric Network and Impact on Swelling. Chim. Techno Acta.

[48] Olăreț E., Voicu Ș.I., Oprea R., Miculescu F., Butac L., Stancu I.C., Serafim A. (2022). Nanostructured Polyacrylamide Hydrogels with Improved Mechanical Properties and Antimicrobial Behavior. Polymers.

[49] Porwal S., Diwedi A., Kamal M. (2012). ^13^C NMR and Raman Studies of Fullerene-Based Poly (Acrylamides). Int. J. Org. Chem..

[50] Wu W., Wu X., He M., Yuan X., Lai J., Sun H. (2021). A novel carbon dot/polyacrylamide composite hydrogel film for reversible detection of the antibacterial drug ornidazole. RSC Adv..

[51] Abo-Zahra S.F., Abdelmonem I.M., Siyam T.E., El-Masry A.M., Abdel-Aziz H.M. (2022). Radiation synthesis of polyacrylamide/functionalized multiwalled carbon nanotubes composites for the adsorption of Cu(II) metal ions from aqueous solution. Polym. Bull..

[52] El-Sweify F.H., Abdelmonem I.M., El-Masry A.M., Siyam T.E., Abo-Zahra S.F. (2019). Adsorption Behavior of Co(II) and Eu(III) on Polyacrylamide/Multiwalled Carbon Nanotube Composites. Radiochemistry.

[53] Cheng M.-M., Huang L.-J., Wang Y.-X., Zhao Y.-C., Tang J.-G., Wang Y., Zhang Y., Hedayati M., Kipper M.J., Wickramasinghe S.R. (2019). Synthesis of graphene oxide/polyacrylamide composite membranes for organic dyes/water separation in water purification. J. Mater. Sci..

[54] Yu S., Li N., Higgins D., Li D., Li Q., Xu H., Spendelow J.S., Wu G. (2014). Self-Assembled Reduced Graphene Oxide/Polyacrylamide Conductive Composite Films. ACS Appl. Mater. Interfaces.

[55] Gayathri K., Palanisamy N. (2020). Methylene blue adsorption onto an eco-friendly modified polyacrylamide/graphite composites: Investigation of kinetics, equilibrium, and thermodynamic studies. Sep. Sci. Technol..

[56] Xie S., Chen Y., Guo Z., Luo Y., Tan H., Xu L., Xu J., Zheng J. (2021). Agar/carbon dot crosslinked polyacrylamide double-network hydrogels with robustness, self-healing, and stimulus-response fluorescence for smart anti-counterfeiting. Mater. Chem. Front..

[57] Suslova E.V., Ulyanov A.N., Kozlov A.P., Shashurin D.A., Savilov S.V., Chelkov G.A. (2023). Composition and Electronic Structure of La_2_O_3_/CNFs@C Core-Shell Nanoparticles with Variable Oxygen Content. Nanomaterials.

[58] Kozlov A., Suslova E., Maksimov S., Isaikina O., Maslakov K., Shashurin D., Savilov S., Shelkov G. (2023). The Preparation of Nanocomposite with a Core–Shell Structure Made of Carbon Matrices and Lanthanum Nanoparticles. Phys. Part. Nucl. Lett..

[59] Suslova E.V., Kozlov A.P., Shashurin D.A., Maximov S.V., Maslakov K.I., Savilov S.V. (2024). La_2_O_3_-carbon composite with core–shell structure and features of its gas-phase oxidation. Mendeleev Commun..

[60] Sivtsov E.V., Kalinin A.V., Gostev A.I., Smirnov A.V., Agibalova L.V., Shumilov F.A. (2020). In Situ Preparation of Polymer Nanocomposites Based on Sols of Surface-Modified Detonation Nanodiamonds by Classical and Controlled Radical Polymerization. Polym. Sci. Ser. B.

[61] Hamon M.A., Chen J., Hu H., Chen Y., Itkis M.E., Rao A.M., Eklund P.C., Haddon R.C. (1999). Dissolution of Single-Walled Carbon Nanotubes. Adv. Mater..

[62] Sudesh, Kumar N., Das S., Bernhard C., Varma G.D. (2013). Effect of graphene oxide doping on superconducting properties of bulk MgB_2_. Supercond. Sci. Technol..

[63] Brusko V., Khannanov A., Rakhmatullin A., Dimiev A.M. (2024). Unraveling the infrared spectrum of graphene oxide. Carbon.

[64] Savilov S.V., Ivanov A.S., Chernyak S.A., Kirikova M.N., Ni J., Lunin V.V. (2015). Features of the oxidation of multiwalled carbon nanotubes. Russ. J. Phys. Chem..

[65] Il’ves V.G., Sokovnin S.Y., Uporov S.A., Zuev M.G. (2013). Properties of the amorphous-nanocrystalline Gd_2_O_3_ powder prepared by pulsed electron beam evaporation. Phys. Solid State.

[66] Gayathri T., Kumar R.A., Dhilipkumaran S., Jayasankar C.K., Saravanan P., Devanand B. (2019). Microwave-assisted combustion synthesis of silica-coated Eu:Gd_2_O_3_ nanoparticles for MRI and optical imaging of cancer cells. J. Mater. Sci. Mater. Electron..

[67] Li J.P.H., Zhou X., Pang Y., Zhu L., Vovk E.I., Cong L., van Bavel A.P., Li S., Yang Y. (2019). Understanding of Binding Energy Calibration in XPS of Lanthanum Oxide by In Situ Treatment. Phys. Chem. Chem. Phys..

[68] Uranta K.G., Rezaei-Gomari S., Russell P., Hamad F. (2018). Studying the Effectiveness of Polyacrylamide (PAM) Application in Hydrocarbon Reservoirs at Different Operational Conditions. Energies.

[69] Gaabour L.H. (2017). Spectroscopic and thermal analysis of polyacrylamide/chitosan (PAM/CS) blend loaded by gold nanoparticles. Res. Phys..

[70] Xu S., Wang Y., Hu J., Liu Z. (2016). Atomic Understanding of the Swelling and Phase Transition of Polyacrylamide Hydrogel. Int. J. Appl. Mech..

[71] Dehkordi N.K., Shojaei S., Asefnejad A., Hassani K., Benisi S.Z. (2023). The effect of three types of cross-linked hydrogels and volume fraction of polyacrylamide on the swelling and thermal behavior using molecular dynamics simulation. J. Mater. Res. Technol..

[72] Sawall S., Amato C., Klein L., Wehrse E., Maier J., Kachelrieß M. (2021). Toward molecular imaging using spectral photon-counting computed tomography?. Curr. Opin. Chem. Biol..

[73] Hemmer E., Yamano T., Kishimoto H., Venkatachalam N., Hyodo H., Soga K. (2013). Cytotoxic aspects of gadolinium oxide nanostructures for up-conversion and NIR bioimaging. Acta Biomater..

[74] Savilov S.V., Strokova N.E., Ivanov A.S., Arkhipova E.A., Desyatov A.V., Hui X., Aldoshin S.M., Lunin V.V. (2015). Nanoscale Carbon Materials from Hydrocarbons Pyrolysis: Structure, Chemical Behavior, Utilisation for Non-Aqueous Supercapacitors. Mater. Res. Bull..

[75] Shabadrov P.A., Safronov A.P. (2018). Superswelling of Hydrogels Based on the Copolymer of Acrylamide and Methacrylic Acid. Polym. Sci. Ser. A.

[76] Neamtu I., Chiriac A.P., Nita L.E. (2006). Characterization of poly(acrylamide) as temperature-sensitive hydrogel. J. Optoelectron. Adv. Mater..

[77] Shen J., Yan B., Li T., Long Y., Li N., Ye M. (2012). Study on graphene-oxide-based polyacrylamide composite hydrogels. Compos. Part A Appl. Sci. Manuf..

[78] Thakur S., Arotiba O. (2018). Synthesis, Characterization and Adsorption Studies of an Acrylic Acid-Grafted Sodium Alginate-Based TiO_2_ Hydrogel Nanocomposite. Adsorpt. Sci. Technol..

[79] Marsh J.F., Jorgensen S.M., Rundle D.S., Vercnocke A.J., Leng S., Butler P.H., McCollough C.H., Ritman E.L. (2018). Evaluation of a photon counting Medipix3RX cadmium zinc telluride spectral X-Ray detector. J. Med. Imaging.

