# Supplementary material for: Composite Contrast Enhancement of Hydrogel-Based Implants for Photon-Counting Computed Tomography Studies
Source: Gels. 2024 Dec 8;10(12):807. doi: 10.3390/gels10120807 (PMC11675360; doi:10.3390/gels10120807)
Supplement: Supplementary file 1 [file gels-10-00807-s001.zip › gels-3335653-supplementary.pdf]

Supplementary Materials

Composite Contrast Enhancement of Hydrogel-Based Implants for Photon-Counting Computed Tomography Studies

Evgeniya V. Suslova [1,*], Denis A. Shashurin [2,*], Konstantin I. Maslakov [1], Stepan Yu. Kupreenko [1], Tatyana O. Luneva [1], Oleg S. Medvedev [2,3] and Georgy A. Chelkov [4]

[1] Department of Chemistry, Lomonosov Moscow State University, Leninskie Gory 1 Bld. 3, 119991 Moscow, Russia; kupreenko@physics.msu.ru (S.Y.K.); luneva.tatiana2016@yandex.ru (T.O.L.)
[2] Faculty of Medicine, Lomonosov Moscow State University, Lomonosovsky Ave. 27 Bld. 10, 119991 Moscow, Russia; oleg.omedvedev@gmail.com
[3] Research and Educational Resource Center for Immunophenotyping, Digital Spatial Profiling and Ultrastructural Analysis Innovative Technologies, RUDN University, 6 Miklukho-Maklaya St., 117198 Moscow, Russia
[4] Joint Institute for Nuclear Research, Joliot-Curie 6, 141980 Dubna, Russia; chelkov@jinr.ru
* Correspondence: suslova@kge.msu.ru (E.V.S.); shashurin@mail.ru (D.A.S.)

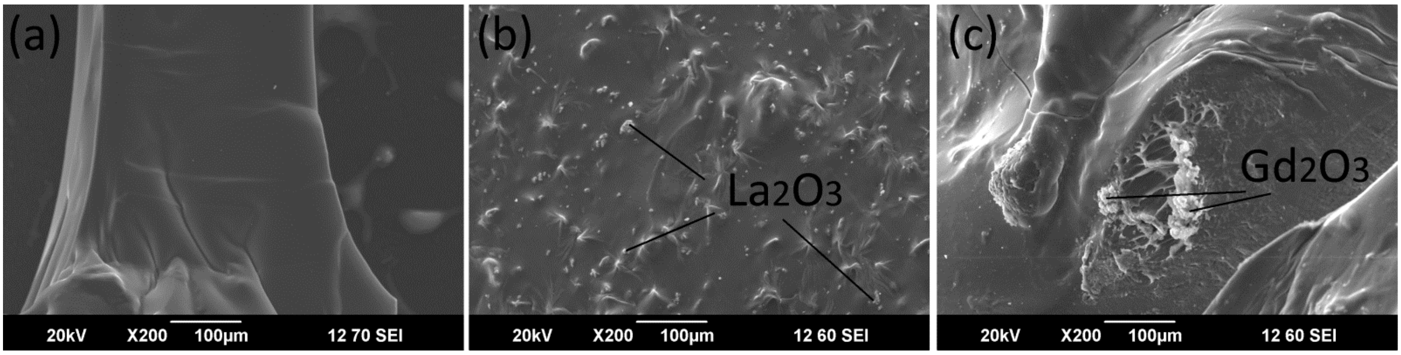

Figure S1. SEM images of PAM (**a**), mixture of La_2O_3 (**b**) and Gd_2O_3 (**c**) with PAM synthesized during polymerization of acrylic acid and acrylamide in the presence of Ln_2O_3.

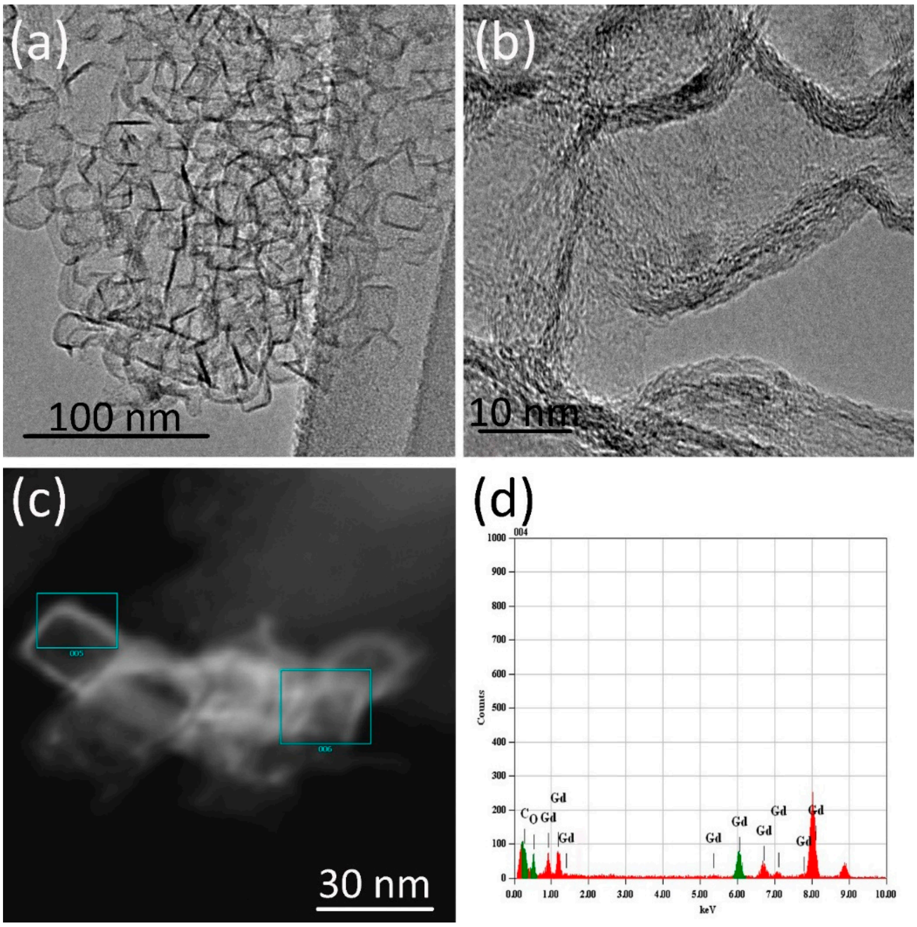

Figure S2. HRTEM (**a**, **b**), HAADF-STEM (**c**) images with EELS spectra of $(Gd_2O_3/GNFs)@C\text{-}COOCH_2C_2H_3$ (**d**).

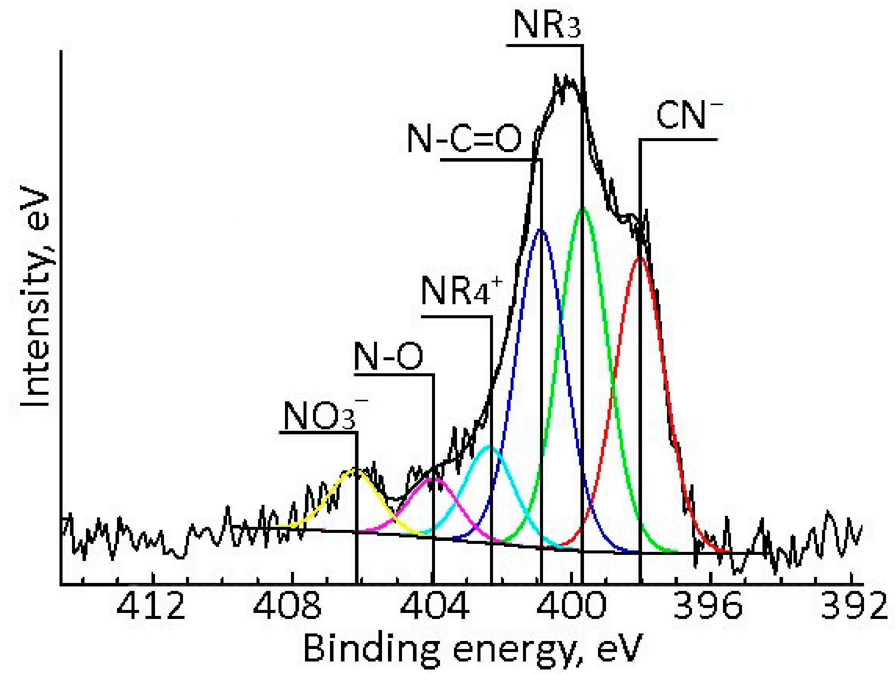

Figure S3. N1s XPS spectra of $(Gd_2O_3/GNFs)@C\text{-}COOCH_2C_2H_3$.
